# The Vessels Shaping Mental Health or Illness

**DOI:** 10.2174/1874205X01913010001

**Published:** 2019-02-15

**Authors:** Jugajyoti Baruah, Anju Vasudevan

**Affiliations:** 1 Department of Psychiatry, Harvard Medical School, Boston, MA-02215, USA; 2 Angiogenesis and Brain Development Laboratory, Division of Basic Neuroscience, McLean Hospital, 115 Mill Street, Belmont, MA-02478, USA

**Keywords:** Brain, Blood vessel, Endothelial cell, Mental health, Psychiatric disorders

## Abstract

The mammalian brain receives the lion’s share of the body’s blood supply and is a highly vascularized organ. The vascular and nervous systems arise at two distinct time points of embryogenesis; however, their functions tend to overlap or complement each other in the growth promoting milieu of the embryonic Central Nervous System (CNS). The pre-existing idea that mental disorders are a direct result from defects solely in neuronal populations and networks is gradually changing. Several studies have implicated blood vessel pathologies and blood flow changes in mental health disorders. Our own studies provide new perspectives as to how intrinsic defects in periventricular endothelial cells, from the earliest developmental time points can lead to the origin of mental health disorders such as schizophrenia, autism spectrum disorders (ASD), anxiety, and depression, thereby establishing direct links. In this article, we provide an overview of how the endothelial cell compartment in the brain is now gaining attention in the context of mental health disorders.

## 1. INTRODUCTION

**Fig. (1).**
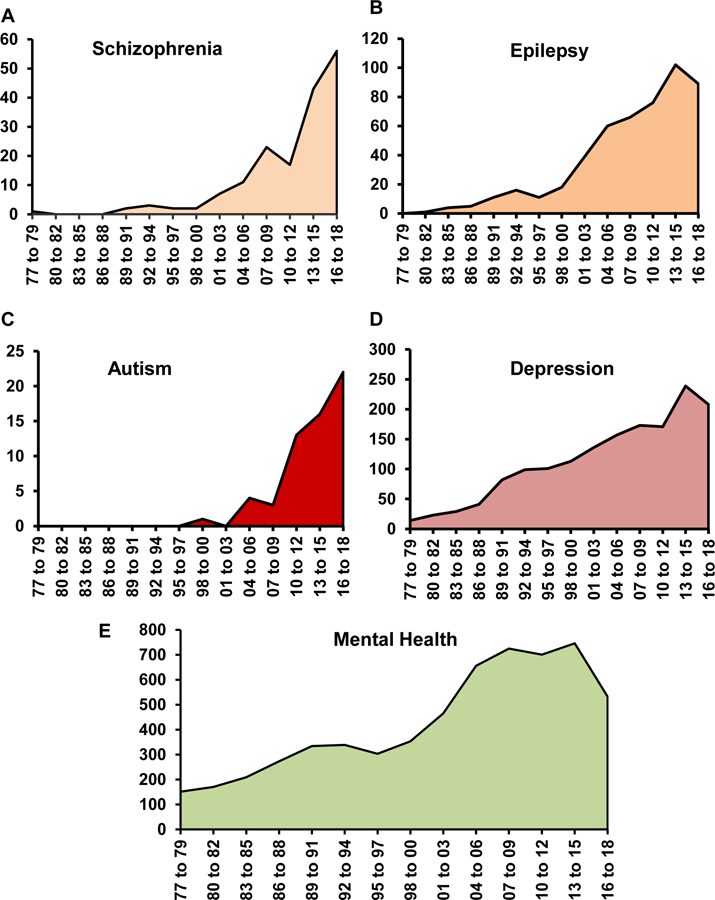
An increasing trend observed in the numbers of endothelial related publications in A) Schizophrenia Β) Autism C) Depression D) Anxiety and E) Mental Health. The PubMed database was searched from January 01^st^, 1977 to December 31^st^ for each consecutive year until September 2018. All articles that were selected by the search hit were included.

The most recent report by *The Lancet Commission* highlighted the global burden of mental health disorders revealing staggering numbers and a prediction for a rapid increase in the number of individuals with a mental health problem [1,2]. It is currently known that an estimate of 1 in 4 people worldwide is affected by one or the other form of mental disorder [3]. Mental health disorder can manifest as schizophrenia, autism spectrum disorder (ASD), anxiety or depression. Most of these illnesses affect multiple functions of the brain and are only partially responsive to therapeutic interventions or pharmacological treatments. The pathological processes involved in the emergence of the phenotype have mostly been ‘neurocentric’. This neurocentric focus implies that defects or malfunctioning in the biology of neurons includes but not limited to neurotransmitter system dys-functions; primarily the Gamma Aminobutyric Acid (GABA)-ergic, dopaminergic or glutamatergic systems, myelin, immune response, infectious reagents or idiopathic reasons [4,5]. However, none of these have accounted for the heterogeneous array of symptoms displayed by the affected individual, thereby making therapeutic intervention only partially successful. But, over the years, this ‘neurocentric’ idea is gradually shifting focus to the contribution of non-neuronal cells, such as the Endothelial Cells (ECs) lining the blood vessels. In a span of 40 years, there has been a sparse yet tangential increase in the number of reports documenting the role of blood vessels in the pathophysiology of mental health disorders (Fig. 1).

As the number of studies highlighting a potential correlation between blood vessel changes in mental health disorders begins to increase, and we know that there is a cross-talk between the developing vasculature and neuronal populations, it is important to question whether defects in the blood vessel system are a *cause* or an *effect* in the etiology of mental health disorders? Here we provide a concise overview of studies reporting defects in brain vascular beds that might have long term implications in the proper functioning and physiological activities of the brain. We describe new conceptual advances that primary defects in the blood vessels of the developing brain can act directly to cause a mental health condition. A greater understanding of the macro and micro vascular alterations in mental health disorders at different stages of life will serve to initiate the use of vascular units as potential therapeutic targets in the future.

### 1.1. A Historical Overview

Prior to the 18^th^ century, an individual presenting signs of ill mental health had to undergo different forms of social and physical abuse. At the turn of the 18^th^ century or the ‘Enlightenment Era’, psychological disorders were commonly referred to as the ‘nervous disorders’ and ‘neurosis’, meaning, a disorder primarily associated with the nervous system. While this paved the way to define a biological as opposed to a supernatural or psychological cause for the symptoms associated with a mental health disorder, it took another century for the physicians, and the psychologists, to comprehend that mental health disorders are not a direct consequence of a malfunctioned nervous system. Given the diagnostic complexities, high comorbidity, and indistinct boundaries that define mental health disorders, it became increasingly evident that the symptoms associated with mental health disorders are multifactorial and not just neurocentric. Among such factors, the foremost is the cellular heterogeneity of the mammalian brain, which in part is responsible for the complex array of symptoms in patients with mental health disorders. The mammalian brain in highly heterogeneous at the cellular level and has a significant percentage of non-neuronal cells [6]. Among the non-neuronal cell population, a major regulator of brain homeostatic function as well as forming the blood-brain barrier (BBB) system is the blood vessels formed of Endothelial Cells (ECs).

Historically, the blood vessels were thought to serve as conduits to deliver nutrients and oxygen to the target tissues, including the brain [7]. Because the brain is a metabolically active organ and is dependent on the blood vessels for its metabolic needs, it is very important that the proper foundation of the brain vasculature is laid during the embryonic stages. An improper patterning of the developing blood vessels can imprint long lasting effects on the proper functioning of the brain and therefore the individual [8,9]. This process of vascularization is accomplished by a well-coordinated, spatiotemporal multistep process called angiogenesis. Angiogenesis is a process whereby ECs proliferate and migrate, to establish vascular networks in target tissues [10,11]. In the context of the mammalian brain, angiogenesis is classified as developmental, physiological or pathological [9]. Developmental angiogenesis orchestrates the spatiotemporal arrangement of the complex CNS vascular networks [12]. Physiological angiogenesis occurs during embryonic development as well as during postnatal growth of the individual to accommodate the growing energy needs. Pathological angiogenesis primarily occurs during adulthood triggered by certain insults/injury, malignant transformation or idiopathic reasons that activate ECs.

**Fig. (2).**
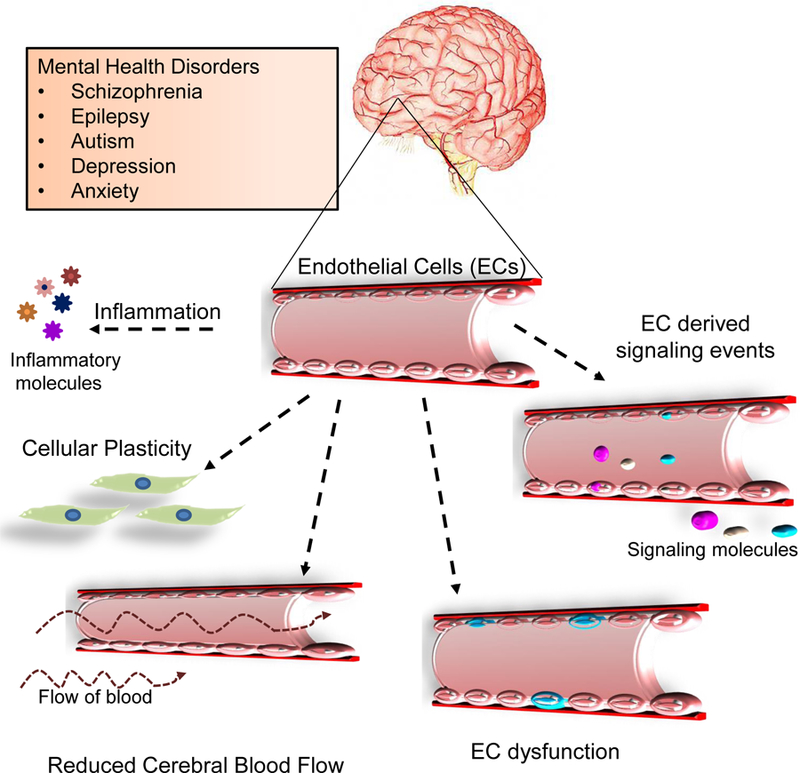
Schema highlighting vascular processes affected in mental health disorders. The brain vascular network which is comprised of endothelial cells can undergo perturbations at multiple levels such as response to inflammatory molecules in circulation, altered plasticity depending on the cellular environmental conditions or changes in the cerebral blood flow (hypoperfusion). Others such as EC dysfunction or EC-derived signaling events can also give rise to disease phenotypes.

In this context, the earliest evidence suggesting observations of vascular abnormalities in the pathophysiology of a mental disorder such as Schizophrenia (SZ) was demonstrated by the work of Bleuler *et al*. utilizing post mortem brain samples [13]. Following this idea, a significant amount of work has been conducted to explain and unify physiological abnormalities of the brain vasculature in other mental disorders (Fig. 2). In addition to defects in angiogenesis, a second common occurrence associated with mental disorders is the disruption of the Blood Brain Barrier (BBB) system. The BBB is an excellent barrier protecting the brain from toxic metabolites, infectious agents and thereby regulating brain homeostasis. At the forefront, ECs are a vital component of the BBB, which have also been studied in the context of mental health disorders. Seminal findings focused on alterations in the BBB came from a simple and small study conducted on veterans that displayed symptoms associated with ill mental health [14]. The patients were administered trace amounts of ions, and later the cerebrospinal fluid and blood were withdrawn to measure the amount of distribution. Alterations in the permeability of the BBB was observed and it was postulated that it might be a ‘metabolic defect’ [14]. But, it was unclear whether changes in the BBB permeability led to symptoms of the psychotic disorders. Thereafter, with improved imaging techniques such as Magnetic Resonance Imaging (MRI), Positron Emission Tomography (PET), and application of cellular and molecular biology techniques, several different hypotheses were proposed regarding the BBB physiology in mental health disorders. Below, we provide a brief summary of different reports that highlight a potential link between changes in blood vessel function per se angiogenesis and/or BBB alterations along with molecular evidence in support of different mental health disorders.

### 1.2. Blood Vessel Dysfunction in Schizophrenia and Epilepsy

Schizophrenia (SZ) is one of the most debilitating mental health disorders and the etiology has remained largely elusive. Following the foundation work by Bleuler *et al*. [13], researchers have tried to focus on understanding the complex interactions between angiogenic factors and neurotrophic factors during development, alterations in blood vessels structure and function, as well as the changes in the BBB. Utilizing the Xenon Xe-133 inhalation method or PET scans, it was observed that most of them displayed hypofrontality, with abnormalities in the hemodynamics of the brain [15,16]. This hypofrontality is consistent with frontal hypoperfusion, meaning, reduced number of vessels in the frontal cortex, which is seen in various regions of the brain. Interestingly, inflow-based-vascular-space-occupancy (iVASO) MRI imaging evaluations have further shown that vessels on the pial surface and arterioles have significant microvascular anomalies and are widespread in the entire brain of a schizophrenic patient [17]. It is postulated that this hypoperfusion accounts for a reduced Cerebral Blood Flow (rCBF), which has been associated with psychotic symptoms [18]. We believe that a reduced vessel number can stem from defects in angiogenesis during early developmental stages, that might result in impaired branching or vessel maturation, and may serve as a possible explanation for the reduced CBF associated with the brains of SZ patients. In parallel, to support the contributions of BBB disruption in the etiology of SZ, post mortem morphometric analyses of prefrontal and visual cortices of SZ individuals showed the presence of ultrastructural abnormalities of capillaries such as thickening of the basal lamina and cytoplasmic vacuolation of ECs [19]. At the molecular level, pathway analysis revealed that most of the well-established angiogenesis-related pathway genes such as Wnt, VEGF, IGF-1, Oncostatin-M, angiopoietin, ephrin-receptor signaling *etc*., were downregulated in the brain regions of individuals with SZ [18].

Another important consideration in SZ is the co-symptomatic presence of epilepsy. Blood vessel changes in the etiology of temporal lobe epilepsy were first demonstrated in a morphological study utilizing surgically resected tissue samples by *Hamada et al*. in 1976 [20]. A decade later, ultrastructural analysis of the BBB showed an increased micropinocytosis in the capillaries during seizures [21]. These were one of the many earlier evidences indicating alterations in the vasculature, especially in the BBB in human psychomotor epilepsy. An increase in interendothelial junctions localized to the seizure-specific regions in the brain was also observed. With the availability of advanced tools and imaging techniques, and a continued interest in vascular contribution towards epilepsy, studies were conducted that highlighted the role of vascular remodeling and angiogenic processes in temporal lobe epilepsy [22,23]. Multiple molecular and cellular mechanisms have been proposed to be involved in the emergence of epilepsy [24,25]. In this context, Vascular Endothelial Growth Factor (VEGF) and its receptors have been studied extensively, highlighting potential mechanisms of how VEGF/VEGFR2 pathway leads to a disruption of BBB in epilepsy [26,27]. VEGF is a key signaling molecule and secreted growth factor in the central nervous system where it mediates angiogenesis, neuroprotection, neuronal survival, and axonal outgrowth. One of the proposed mechanisms involving VEGF is that an epilepsy episode leads to an increased expression of VEGF, which in turn promotes angiogenesis, and increases vascular permeability. Another important pathway implicated in epilepsy is the Ephrin-A5/EphrinA4 pathway, which modulates angiogenesis via the activities of phosho-Akt and phosho-ERK [28].

In addition to this, studies conducted using chemo-convulsive rat models administered with kainate and pilocarpine emphasized a strong correlation between vascular damage and astroglial function. Astrocytic lesions in the specific *Cornu Ammonis 3* (CA3) and Piriform (Pir) cortex regions of the brain were significantly related to blood vessel damage in these areas [29,30]. Especially in the CA3 region, this damage and subsequent remodeling may deeply affect the hippocampal response to inputs from dentate gyrus and entorhinal cortex. This damage was accompanied by micro-hemorrhages and loss of vessel integrity that is often seen in cerebral ischemia leading to hypoxic conditions. Such studies once again underscore the importance of vascular function in the pathophysiology of epilepsy [29,30]. Through this evidence, one may theorize that vascular protection during epilepsy may ameliorate the ischemic effects in specific brain regions. To this end, a recent study conducted by *Vinet et al*. showed that experimental use of a matrix metalloprotease (MMP) inhibitor-12 limited ischemic-like lesion formation in the hippocampus of a pilocarpine-induced rat model of epilepsy by reducing BBB leakage at the lesion site [31]. Even though the studies till date have improved our understanding of vascular changes in mental health disorders, there are significant and important questions that remain to be addressed.

### 1.3. Blood Vessel Dysfunction in Autism Spectrum Disorders (ASD)

Herold S. measured the regional cerebral blood flow along with oxygen and glucose consumption using PET imaging in young autistic adults. While this was the first kind of a study trying to link blood vessel function such as cerebral blood flow (CBF) to autism, he was not able to report any significant differences between healthy controls and autistic individuals [32]. Later, an extensive work conducted by Zilbovicius *et al*., utilized Single Photon Emission Computed Tomography (SPECT) imaging and observed a potential link between delayed CBF in the frontal lobe of autistic children [33]. These studies showed that delayed metabolic maturation of the frontal lobes arising from hypoperfusion might constitute an important link between cognitive defects and brain dysfunction in childhood autism. Due to this hypoperfusion, the neuronal connections are altered in the brain affecting overall neural network formation and brain development. One of the many causes for this reduced blood flow to the cerebral cortex was proposed as endothelial inflammation or endothelial dysfunction. Another important correlation was the expression of vascular endothelial growth factor (VEGF) and its endogenous antagonists, soluble VEGF receptors (sVEGFR)-1 and −2. In subjects with severe ASD, a lower level of VEGF and increased sVEGFR-1 in the serum were reported [34]. This reduced expression of VEGF can account for a decrease in angiogenesis, therefore reduced number of vessels or hypoperfusion of the brain, which supports the link between decreased cerebral blood flow and ASD. Additionally, expression levels of serum platelet endothelial cell adhesion molecule (PECAM)-1, soluble vascular cell adhesion molecule-1 (sVCAM-1) and soluble P-selectin were significantly reduced in children with ASD [35,36]. Interestingly, a more recent immunocytochemical staining of post-mortem brain slices has revealed persistent angiogenesis in the child and young adult patients with ASD, where blood vessels are in constant flux rather than expanding [37] and highlights that blood vessel plasticity is a global component of the ASD brain. In this study, the authors propose that repetitive seizures, a common symptom associated with ASD patients, cause an increase in the brain activity which then leads to splitting or intussusceptive angiogenesis that further guides neuronal rearrangement. This rearrangement of the microvasculature allows for the excessive and shorter local connectivity within the neurons, which then prevents the growth of longer and more complex brain connections required for language and social interactions. In a small yet significant study, *Bashir* & *Ayadhi* [2015] reported an increase in anti-endothelial antibody levels in the sera of children with autism [38]. Although studies as such will require validation with a larger cohort and with molecular methodologies, the alteration of the blood vessel environment in ASD is evident.

### 1.4. Blood Vessel Dysfunction in Depression and Anxiety

In an attempt to highlight the importance of blood vessels in depression, Alexopoulus *et al*. proposed the “Vascular Depression hypothesis” that summarized clinical studies of how patients with vascular disease such as hypertension, ischemic heart disease, peripheral vascular disease, stroke, heart failure, coronary artery disease and vascular dementia, often have depression [39] and questioned whether the damage of vascular structures contributed to depression in geriatric patients? Today, as we know, depression affects an individual irrespective of age. As investigators continue to understand the biological basis of depression, it is clear that blood vessel health is of significance in people suffering from depression. Importantly, the phenotypes associated with depression are multivariate, and therefore, there exists a causal relationship between depression and anxiety. The understanding of how dysfunction of the blood vessel component might be important in these two forms of psychological disorders is very recent compared to other extensively studied forms of mental health disorders.

An interesting feature of depression related disorder and its subtypes is that, often times, depression is more prevalent in patients with Coronary Artery Disease (CAD) [40], where patients exhibit abnormal endothelial function. Several post mortem analyses of the orbitofrontal cortex and other regions of the elderly patients with major depressive disorder (MDD) showed morphological changes in the vascular structure, specifically an increase in perivascular spaces and increased intracellular cell adhesion molecule-1 (ICAM-1) [41,42]. Importantly, a correlative study using a rat model of depression revealed impaired function of the endothelium-derived hyper-polarizing (EDH)-like relaxation factor in small resistance arteries [43]. At the molecular level, the expression of several key endothelial cell molecules such as IGF1, VEGF, VEGF-A is altered in patients with depression [44,45]. Moving forward from the vascular depression hypothesis, a most recent review by Burrage *et al.* clearly highlight the potential role of cerebrovascular dysfunction in depression, along with some key molecules such as VEGF, nitric oxide (NO), and thrombospondin-1 (TSP-1), that might have implications in exacerbating the phenotype [46]. It was found that patients exhibiting subclinical depressive status without any evident CVD had a low number of circulating endothelial progenitor cells (EPC) or an altered vascular function [47,48]. In addition, using high resolution imaging of the retinal vasculature, certain associations have been made between the retinal micro-vasculature and/or endothelial function, such as flow-mediated dilation in young and adolescent patients, where the authors propose that alterations of the microvasculature at an early age predispose individuals to develop either depression and anxiety [49,50]. Another important criterion of endothelial cell function such rCBF was significant in various areas of the brain [51]. As it is a well-established phenomenon that hypoperfusion leads to psychomotor retardation and impaired executive function, therefore it is not surprising that an impairment of blood vessel function is seen during depression and/or anxiety. At the molecular level, activation of GABAB2 which increases cerebral hypoperfusion leads to the development of anxiety-like behaviors in a rodent model of cerebral ischemia [52].

Together, all these studies provide insights into alterations in vascular pathology, disturbances in cerebral blood flow or abnormalities in blood vessel function in post-mortem samples, rodent models and patients with the use of old and new technologies. Several of these vascular disturbances are linked to inflammation or changes in neural plasticity at various stages of life. We have summarized the various vascular abnormalities reported in mental disorders into a schema (Fig. 2). However, these studies do not clearly implicate abnormalities in vessels as a direct cause for the disease. Recent studies in our laboratory provide new insights into this direction.

### 1.5. The Vascular Origin of Mental Health Disorders

**Fig. (3).**
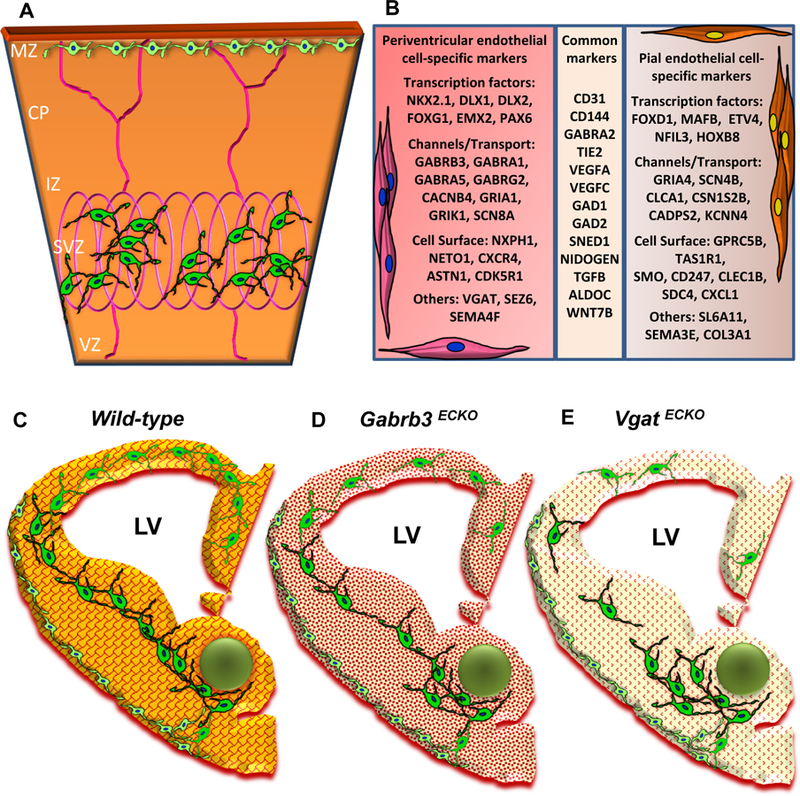
(A) Schema depicting selective sorting of superficial stream of GABAergic interneurons in the MZ by pial vessels and deep stream of GABAergic interneurons in the SVZ by the periventricular vessels of the embryonic telencephalon [E13]. (B) A table depicting some important markers that distinguish periventricular endothelial cells from pial endothelial cells in the embryonic telencephalon. Common markers expressed in pial and periventricular endothelial cells are also shown. (C-E) Schema depicting conceptual changes in GABA signaling during brain development. (C) Wildtype embryonic telencephalon with normal periventricular vascular network (red lattice pattern) and normal endothelial GABA signaling pathway (reddish-orange hue) promotes tangential GABAergic neuronal migration (green) from the ventral telencephalon. (D) In *Gabrb3* ^*ECKO*^ telencephalon, which has dysfunctional endothelial GABA_A_ receptors, there is a partial loss of endothelial GABA secretion (light orange hue). This affects periventricular angiogenesis (intricate red pattern) and neuronal migration with a reduction in GABAergic interneurons in the developing neocortex. (E) In *Vgat* ^*ECKO*^ telencephalon, there is a complete loss of endothelial GABA secretion (light yellowish hue) that affects periventricular angiogenesis (dotted red pattern) with more significant consequences for GABAergic neuronal migration resulting in abnormal cortical distribution.

In the last decade, our work has made significant conceptual advances with respect to CNS angiogenesis from developmental and disease perspectives [53 – 56]. The change began with the finding that blood vessels in the embryonic forebrain (telencephalon) are not just a homogenous population of vessels, responding passively to the metabolic demands of growing neuronal populations [53]. Based on anatomy, origin, gene expression patterns and developmental mechanisms, there are two distinct telencephalic vascular networks pial and peri-ventricular. The tapering vessels joining the periventricular and pial vessels may represent the earliest arterial-venous communication. The periventricular vascular network develops in advance of and independent of neuronal development by embryonic day 11 to act as a substrate and provide critical guidance cues to instruct key events that follow in the embryonic telencephalon, for instance, neurogenesis, radial migration of projection neuron precursors and tangential migration of GABAergic interneurons [54,55]. The pial and periventricular vascular networks provide separate guidance cues to the superficial stream of GABAergic interneurons in the Marginal Zone (MZ) versus the deep stream of GABAergic neurons in the Subventricular Zone (SVZ) during long distance tangential neuronal migration (Fig. 3A). We have summarized important markers that are specific to periventricular endothelial cells versus pial endothelial cells and common markers that identify both cell types (Fig. 3B). Interestingly, the periventricular vascular network not only acts as a physical substrate for neuronal migration but also it holds the key to several novel developmental mechanisms and pathways [54 – 56]. Gene expressions for biological processes and canonical maps containing genes controlling neurogenesis, neuronal migration, chemotaxis, and axon guidance were enriched in periventricular endothelial cells when compared to pial endothelial cells. For instance, genes commonly known to be expressed and/or traditionally believed to be confined to GABAergic neurons/interneurons and their precursors were found to be expressed/even upregulated in periventricular endothelial cells. Pial endothelial cells, on the other hand, showed enrichment in inflammation and pathological process categories. When genes expressed in periventricular endo-thelial cells were classified according to disease categories, an enrichment was observed in psychiatric disease categories [55]. Our studies thus implicated a new cellular substrate periventricular endothelial cells as being a contribution to a wide swath of mental health diseases with schizophrenia, epilepsy, bipolar, mood, depressive disorders and autism topping the list [55]. These results also highlighted the great need to validate and understand the functional significance of novel genes expressed in periventricular endothelial cells/blood vessels and its specific contribution to psychiatric disease symptoms.

This, however, was not an easy task. All the mouse models reported until now are complete/systemic or region specific knockouts of the GABA_A_ receptor-GABA pathway making it difficult to establish a cause-effect relationship between endothelial and neuronal development and cell-type specific contributions to the origin of neuropsychiatric illness. To discover the functional significance of the endothelial GABA signaling pathway from early developmental stages onward, we designed strategies to specifically render endothelial GABA_A_ receptors dysfunctional (the *Gabrb3* endothelial cell conditional knockout or *Gabrb3* ^*ECKO*^) or turn off GABA release from endothelial cells (the *Vesicular GABA Transporter, Vgat)* endothelial cell conditional knockout or *Vgat* ^*ECKO*^) by using CRE-LOX conditional gene knockout technology [56]. Both approaches markedly affected periventricular angiogenesis throughout prenatal development. Reduction in this vascular substrate, in turn impaired GABAergic neuronal tangential migration, albeit more severely in the *Vgat* ^*ECKO*^ model versus the *Gabrb3* ^*ECKO*^ model (Fig. 3C–E). Concurrent vascular and GABA cell deficits persisted in the postnatal cerebral cortex with significant consequences for postnatal behavior [56]. Partial loss of endothelial GABA release was sufficient to cause behavioral dysfunction similar to the psychiatric disease that is characterized by one or more of these core symptoms - impaired social recognition, reduced social interactions, communication deficits, increased anxiety or depression. Complete loss of GABA release resulted in significant abnormalities in developmental milestones and resulted in a model reminiscent of childhood epilepsy or ASD. Alterations in postnatal behavior were characterized by periods of quiescence, interrupted by tremors and a reduction in voluntary movement and the mice failed to survive beyond 2 months of age. Our studies for the first time, describe how intrinsic defects within telencephalic vasculature/endothelial cells from the earliest developmental time points can independently mold neuronal signaling pathways with far-reaching consequences for brain development and behavior [56]. It also highlights a vascular GABA signaling pathway that is distinct from the neuronal GABA signaling and how variations in vascular GABA levels can cause diversity in psychiatric symptoms.

## CONCLUSION AND PERSPECTIVES

The present review aims to provide an overview of progressive research in understanding the contribution of blood vessel anomalies in mental illness. The advances that took place were sparse and heterogeneous over the past forty years (Fig. 1), which suggest that endothelial cell contribution to the pathophysiology of mental illness was an underappreciated area of research. Most of the initial derivations were based on observations from structural studies and anatomical anomalies in patients exhibiting one or the other form of mental illness. However, over the next several decades, seminal observations by various investigators shifted this idea and emphasized the presence of vascular abnormalities in mental illnesses. Our own studies show how alterations in a novel GABA pathway in endothelial cells can contribute to the origin of mental health disorders [55,56].

It is interesting that exercise and spiritual activities like meditation, are often recommended for improvement of mental health. Both activities have beneficial effects on the brain by triggering angiogenesis and blood flow that in turn improve cognitive performance [57,58]. However, if there is an intrinsic defect within brain blood vessels, such options may not work. A better understanding of the spatiotemporal regulation of EC gene expression and function in brain vascular networks at both developmental and adult stages in normal and disease conditions is critical for the future. We hope that the ‘healing touch’ of angiogenesis therapy that has brought relief to patients with stroke, blinding eye diseases, cancer and neuro-degeneration will gradually extend to the field of mental health disorders over the next years. Determination of brain blood vessel health should become a part of our routine health visits and check-ups.
